# Supplementary material for: Fish Consumption: Influence of Knowledge, Product Information, and Satisfaction with Product Attributes
Source: Nutrients. 2022 Jun 28;14(13):2691. doi: 10.3390/nu14132691 (PMC9269055; doi:10.3390/nu14132691)
Supplement: Supplementary file 1 [file nutrients-14-02691-s001.zip › nutrients-1734019-supplementary.pdf]

Supplementary Materials S1: Questionnaire.

1. *How often do you personally consume following fishery products at home?*

Type of fishery product	Every day	4-5 times a week	2-3 times a week	Once a week	2-3 times a month	Once in 3 months	Once a year or less
White fish (sea bream, sea bass, hake and other)							
Fatty fish (sardine, mackerel, chub mackerel, tuna and other)							

2. *We kindly ask you to indicate if the answer is true or false*

No.	Statements	TRUE	FALSE
1.	Fish is a source of dietary fibre.		
2.	Fish is a source of omega-3 fatty acids.		
3.	It is recommended to eat fish at least twice a week.		
4.	Consumption of fatty fish is important in the prevention of some chronic diseases, such as cardiovascular diseases.		
5.	High maternal fish consumption during pregnancy and infant's fish intake in the first year improves child developmental skills.		
6.	The sea bass and sea bream available in the European market are exclusively wild species.		
7.	The eyes of the fish demonstrate its freshness.		

3. *We kindly ask you to indicate the level of your agreement with the following statements on a scale.*

**No.**	**Statement**	Strongly DISAGREE				Strongly AGREE
1.	I consider that I know more about fish than the average person.	1	2	3	4	5
2.	I think that I know more about fish than my friends.	1	2	3	4	5
3.	I have a lot of knowledge about how to prepare fish.	1	2	3	4	5
4.	I have a lot of knowledge about how to evaluate the quality of fish.	1	2	3	4	5

4. *We kindly ask you to indicate on the scale from 1 to 5 the level of your satisfaction with the following attributes of the fishery products.*

No.	Product attributes	Extremely DISSATISFIED				Extremely SATISFIED
1.	Price	1	2	3	4	5
2.	Quality	1	2	3	4	5
3.	Price - quality ratio	1	2	3	4	5
4.	Availability	1	2	3	4	5
5.	Choice	1	2	3	4	5
6.	Freshness	1	2	3	4	5

5. *When buying fishery products, how important is the following information to you? We kindly ask you to indicate the level of importance on the scale.*

No.	Product information	Not at all IMPORTANT				Very IMPORTANT
1.	Shelf life	1	2	3	4	5
2.	Nutritional value (the content of energy, proteins, fats and other nutrients)	1	2	3	4	5
3.	List of ingredients	1	2	3	4	5
4.	Country of origin	1	2	3	4	5
5.	Production method (wild vs farmed)	1	2	3	4	5
6.	Product brand	1	2	3	4	5
7.	Processing method (canning, special packaging, smoking etc.)	1	2	3	4	5
8.	Quality label	1	2	3	4	5
9.	Eco-label	1	2	3	4	5
10.	Previous freezing	1	2	3	4	5
11.	Recommended method of preparation	1	2	3	4	5

Socio-demographic profile of respondents

1.	Age:	
2.	Sex:	a) Male<br>b) Female

5.	Average household income per month	Croatia<br>a) Less than 5 000 HRK / Less than 667.7 EUR<br>b) 5 001 - 10 000 HRK / 667.8 - 1335.5 EUR<br>c) 10 001 - 15 000 HRK / 1335.6 - 2003.2 EUR<br>d) 15 001 - 20 000 HRK / 2003.3 - 2670.9 EUR<br>e) More than 20 001 HRK / 2671.0 EUR<br>f) Prefer not to say<br>Italy<br>a) Less than 1500 EUR<br>b) 1 501 - 4 000 EUR<br>c) 4 001 - 7 500 EUR<br>d) 7 501 - 10 000 EUR<br>e) More than 10 000 EUR<br>f) Prefer not to say
6.	Your level of education:	a) Primary school or lower<br>b) Secondary school<br>c) Bachelor, master or higher
9.	Region	Croatia:<br>a) Zagreb (City of Zagreb)<br>b) North Croatia (North)<br>c) Slavonia (East)<br>d) Lika and Banovina (Centre)<br>e) Istra, Rijeka and Primorje (South-west)<br>f) Dalmatia (South)<br>Italy:<br>a) Nord-ovest (North-west)<br>b) Nord-est (North-east)<br>c) Centro (Center)<br>d) Sud (South)<br>e) Isole (Islands)
